# Supplementary material for: Asynchronous magnetic resonance elastography: Shear wave speed reconstruction using noise correlation of incoherent waves
Source: Magn Reson Med. 2022 Oct 27;89(3):990–1001. doi: 10.1002/mrm.29502 (PMC9792433; doi:10.1002/mrm.29502)
Supplement: Supplementary file 1 — DATA S1 MATLAB code used to generate the numerical simulations of Figure 1 [file MRM-89-990-s002.zip › k-Wave/helpfiles/cart2grid.html]

cart2grid :: Functions (k-Wave)


# cart2grid

Interpolate a set of Cartesian points onto a binary grid.

## Syntax

```
[grid_data, order_index, reorder_index] = cart2grid(kgrid, cart_data)
```

## Description

`cart2grid` interpolates the set of Cartesian points defined by `cart_data` onto a binary matrix defined by the k-Wave grid object `kgrid` using nearest neighbour interpolation. An error is returned if the Cartesian points are outside the computational domain defined by kgrid.

## Inputs

|  |  |
| --- | --- |
| `kgrid` | k-Wave grid object returned by `kWaveGrid` |
| `cart_data` | 1 x N, 2 x N, or 3 x N (for 1, 2, and 3 dimensions) array of Cartesian sensor points |

## Outputs

|  |  |
| --- | --- |
| `grid_data` | binary grid with the same dimensions as `kgrid` |
| `order_index` | the order that the Cartesian points appear in `grid_data` according to MATLAB's standard column-wise linear matrix index ordering |
| `reorder_index` | the order that the binary points in `grid_data` (according to MATLAB's standard column-wise linear matrix index ordering) appear in the original Cartesian data |

## Examples

- Homogenous Propagation Medium
- Loading External Image Maps
- Comparison Of Modelling Functions
- Simulations In Three Dimensions
- Modelling Sensor Directivity in 2D
- Modelling Sensor Directivity in 3D
- 2D Time Reversal For A Circular Sensor
- 3D Time Reversal For A Spherical Sensor
- Simulations In Three Dimensions
- Optimising k-Wave Performance

## See Also

`grid2cart`, `interpCartData`, `kWaveGrid`
